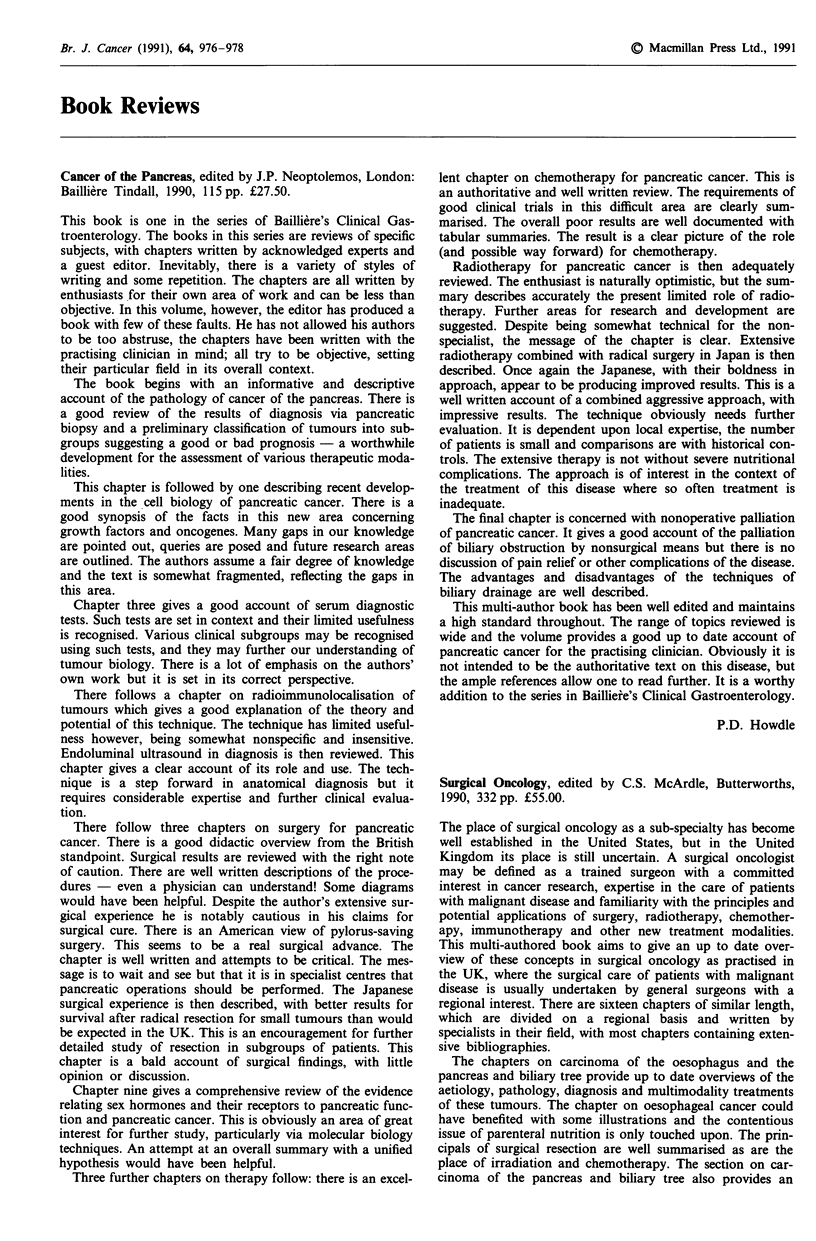# Cancer of the Pancreas

**Published:** 1991-11

**Authors:** P.D. Howdle


Br. J. Cancer (1991), 64, 976–978 © Macmillan Press Ltd., 1991

### Book Reviews

**Cancer of the Pancreas**, edited by J.P. Neoptolemos, London: Baillière Tindall, 1990, 115 pp. £27.50.

This book is one in the series of Baillière's Clinical Gastroenterology. The books in this series are reviews of specific subjects, with chapters written by acknowledged experts and a guest editor. Inevitably, there is a variety of styles of writing and some repetition. The chapters are all written by enthusiasts for their own area of work and can be less than objective. In this volume, however, the editor has produced a book with few of these faults. He has not allowed his authors to be too abstruse, the chapters have been written with the practising clinician in mind; all try to be objective, setting their particular field in its overall context.

The book begins with an informative and descriptive account of the pathology of cancer of the pancreas. There is a good review of the results of diagnosis via pancreatic biopsy and a preliminary classification of tumours into subgroups suggesting a good or bad prognosis — a worthwhile development for the assessment of various therapeutic modalities.

This chapter is followed by one describing recent developments in the cell biology of pancreatic cancer. There is a good synopsis of the facts in this new area concerning growth factors and oncogenes. Many gaps in our knowledge are pointed out, queries are posed and future research areas are outlined. The authors assume a fair degree of knowledge and the text is somewhat fragmented, reflecting the gaps in this area.

Chapter three gives a good account of serum diagnostic tests. Such tests are set in context and their limited usefulness is recognised. Various clinical subgroups may be recognised using such tests, and they may further our understanding of tumour biology. There is a lot of emphasis on the authors' own work but it is set in its correct perspective.

There follows a chapter on radioimmunolocalisation of tumours which gives a good explanation of the theory and potential of this technique. The technique has limited usefulness however, being somewhat nonspecific and insensitive. Endoluminal ultrasound in diagnosis is then reviewed. This chapter gives a clear account of its role and use. The technique is a step forward in anatomical diagnosis but it requires considerable expertise and further clinical evaluation.

There follow three chapters on surgery for pancreatic cancer. There is a good didactic overview from the British standpoint. Surgical results are reviewed with the right note of caution. There are well written descriptions of the procedures — even a physician can understand! Some diagrams would have been helpful. Despite the author's extensive surgical experience he is notably cautious in his claims for surgical cure. There is an American view of pylorus-saving surgery. This seems to be a real surgical advance. The chapter is well written and attempts to be critical. The message is to wait and see but that it is in specialist centres that pancreatic operations should be performed. The Japanese surgical experience is then described, with better results for survival after radical resection for small tumours than would be expected in the UK. This is an encouragement for further detailed study of resection in subgroups of patients. This chapter is a bald account of surgical findings, with little opinion or discussion.

Chapter nine gives a comprehensive review of the evidence relating sex hormones and their receptors to pancreatic function and pancreatic cancer. This is obviously an area of great interest for further study, particularly via molecular biology techniques. An attempt at an overall summary with a unified hypothesis would have been helpful.

Three further chapters on therapy follow: there is an excellent chapter on chemotherapy for pancreatic cancer. This is an authoritative and well written review. The requirements of good clinical trials in this difficult area are clearly summarised. The overall poor results are well documented with tabular summaries. The result is a clear picture of the role (and possible way forward) for chemotherapy.

Radiotherapy for pancreatic cancer is then adequately reviewed. The enthusiast is naturally optimistic, but the summary describes accurately the present limited role of radiotherapy. Further areas for research and development are suggested. Despite being somewhat technical for the non-specialist, the message of the chapter is clear. Extensive radiotherapy combined with radical surgery in Japan is then described. Once again the Japanese, with their boldness in approach, appear to be producing improved results. This is a well written account of a combined aggressive approach, with impressive results. The technique obviously needs further evaluation. It is dependent upon local expertise, the number of patients is small and comparisons are with historical controls. The extensive therapy is not without severe nutritional complications. The approach is of interest in the context of the treatment of this disease where so often treatment is inadequate.

The final chapter is concerned with nonoperative palliation of pancreatic cancer. It gives a good account of the palliation of biliary obstruction by nonsurgical means but there is no discussion of pain relief or other complications of the disease. The advantages and disadvantages of the techniques of biliary drainage are well described.

This multi-author book has been well edited and maintains a high standard throughout. The range of topics reviewed is wide and the volume provides a good up to date account of pancreatic cancer for the practising clinician. Obviously it is not intended to be the authoritative text on this disease, but the ample references allow one to read further. It is a worthy addition to the series in Baillieře's Clinical Gastroenterology.

P.D. Howdle